# Endothelial Dysfunction and Ischemia-Modified Albumin Levels in Males with Diabetic and Nondiabetic Erectile Dysfunction

**DOI:** 10.1155/2022/3661822

**Published:** 2022-05-09

**Authors:** Hasan Anıl Kurt, Emrah Demirci, Cabir Alan

**Affiliations:** ^1^Canakkale Onsekiz Mart University, Medical Faculty, Department of Urology, Turkey; ^2^Yozgat City Hospital, Department of Urology, Turkey

## Abstract

In this study, we aimed to determine endothelial dysfunction and ischemia-modified albumin (IMA) levels in patients diagnosed with erectile dysfunction (ED) and to examine the relationship between these and diabetes disease. 86 male patients (46 patients with diabetes, age: 51.5 ± 9.2 and 40 patients with nondiabetes (control group), age: 54.78 ± 12.2) were included in the study. IMA, a new indicator of tissue ischemia and oxidative stress, was checked. Superoxide dismutase (SOD) activity, another oxidative stress indicator, was examined. Endothelin-1 (ET-1), one of the parameters of endothelial dysfunction, was measured. Additionally, endothelial function was evaluated with flow-mediated vasodilatation (FMD). Student's *t*-test was used for statistical evaluation. *p* values less than 0.05 were considered statistically significant. SOD activity was significantly lower in the diabetic group than in the control group, and ET-1 was significantly higher (*p* < 0.001). IMA was found to be significantly higher in the diabetic group than the control group (*p* < 0.001). FMD was significantly lower in diabetic group compared to the control group (*p* < 0.002). According to our findings, the co-occurrence of erectile dysfunction and diabetes demonstrates a complex condition that includes endothelial dysfunction, oxidative stress, and tissue ischemia. When the correlation of indicators, which are markers, was examined, the severity of the co-occurrence of diabetes and erectile dysfunction was again demonstrated.

## 1. Introduction

Erectile dysfunction (ED) is an important health issue, demonstrated a high prevalence and generally increasing incidence worldwide, and is described as inability to achieve and/or sustain an adequate erection for sexual activity [1, 2]. Many physical factors, including vascular, neurological, and hormonal as well as cavernosal factors and psychological factors, are associated with normal erectile function. An abnormality in one or several of these factors may lead to ED.

Given the results of epidemiological studies, primary risk factor for erectile dysfunction is age. Prevalence and severity of ED increase with age. In the study, it was reported that the most significant risk factor second to age was diabetes [3, 4]. Diabetes is the second most prevalent risk factor for erectile dysfunction. Approximately 50% (between 28% and 59%) of patients with diabetes suffer from ED regardless of the type of diabetes. ED prevalence in diabetics depends on age and severity of diabetes [5].

Diabetes is a systemic disease giving rise to metabolic abnormalities in regulation of mechanism of carbohydrates. Such abnormalities cause pathologies like ophthalmology, neuropathy, nephropathy, and vasculopathy in various organ systems. Diabetic complications can be broken down into two categories, i.e., diabetes-specific (retinopathy, nephropathy, and neuropathy) and nonspecific (vasculopathy and others). Main factors increasing the risk of developing complications are blood glucose level and duration of exposure to hyperglycemia. A number of events occur in DM as hyperglycemia is associated with increased oxidative stress and hyperproduction of reactive oxygen species (ROS). A decrease in NO and an increase in tissue factor and prothrombotic factors such as plasminogen activator inhibitor-1, endothelin-1, subsequent thrombosis, and vasoconstriction, as well as an increase in nuclear factor kappa *β*, result in ED [6]. Glycemic control is a factor associated with ED. Peripheral neuropathy and hemoglobin A1c level are independent risk factors for ED [7, 8].

Besides an advancing age and diabetes, hypertension, hyperlipidemia, smoking, and obesity are also risk factors for ED. In addition to ED, all of these risk factors are also risk factors in common with cardiovascular disease (CVD) [9, 10]. Vascular insufficiency by atherosclerosis is present in the pathophysiology of both ED and CVD caused. The main reason underlying the formation of atherosclerotic plaques is, however, the endothelial dysfunction promoted by oxidative stress [11]. Endothelial dysfunction and penile atherosclerosis are clinical situations in common between ED and diabetes mellitus. The association between these two clinical situations is complicated and may involve other pathophysiological mechanisms such as autonomous neuropathy as well as hormonal changes [12].

Albumin is a protein with different functions, which is abundant in blood, and composed of residues of 585 amino acids, and is synthesized in the liver [13]. In cases such as free radical damage, energy-related membrane destruction, exposure to free iron and copper, acidosis, and hypoxia, N-terminal end of albumin is modified resulting in its capacity to bind to transition metals like Co+2, Ni+2, and Cu+2 [14]. This modified form of albumin is called ischemia-modified albumin (IMA). IMA significantly increases in patients with diabetes and in poor glycemic control and also has a significant relationship with HbA1c [15].

In this study, we aimed to determine endothelial dysfunction and IMA levels in patients diagnosed with ED and evaluate its association with diabetes. When ED is associated with DM, we encounter a severe ED form. This may require complicated treatment. This is the first study, to our knowledge, investigating the relationship between IMA and ED in patients with diabetes.

## 2. Material and Method

This study was run by the Urology Department of Çanakkale Onsekiz Mart University Medical School. The study was approved by the Ethical Board of Çanakkale Onsekiz Mart University Medical School.

Patients over 40 years of age with type 2 diabetes—receiving oral or subcutaneous antidiabetic therapy for at least 1 year—and nondiabetic ED presenting to our urology outpatient clinic were evaluated. Presence of CVD and abnormal testosterone levels was regarded as exclusion criterion. 86 male patients with ED (46 patients with diabetes, age: 51.5 ± 9.2 and 40 patients with nondiabetes (control group), age: 54.78 ± 12.2) were included in the study.

The International Index of Erectile Function-IIEF developed by Rosen et al. was used to evaluate erectile function [16]. According to the scores obtained from questions 1-5 and 15, patients with a score of 10 or less were included in the study.

Blood samples from diabetic and nondiabetic men with ED were centrifuged at 4000 rpm for 10 minutes, and their sera were separated. Sufficient serum samples were separated for biochemical analysis to be studied later and stored at -80°C in the freezer until analysis time.

The method developed by Bar-Or et al. [17] was used in determination of IMA. The test called albumin cobalt binding test (ACB) is based on the binding of serum albumin and cobalt which is a transition metal. A spectrophotometer of Spekol 1300 brand was used for measurements. Results were reported as absorbance units (ABSU).

Superoxide dismutase (SOD) activity measurement, one of the oxidative stress markers, was measured with the Cayman ELISA kit.

Endothelin-1 (ET-1) measurement which is one of the endothelial dysfunction parameters was measured with the Bachem ELISA kit. In addition, endothelial function was tried to be evaluated by flow mediated vasodilation (FMD).

FMD is a simple, repeatable, and safe technique performed along with echocardiography. In FMD technique, generally, the dilation response of the artery (subject to measurement) to the increased blood flow and the resulting increase in stress in the vessel wall is measured [18].

The FMD test was measured by the same cardiologist who was not aware of the clinical or laboratory profile of the patients. The patients were told to be fasted for at least twelve hours and not to drink alcohol and caffeine during this period. Measurements were made at 22-25°C of room temperature in silence. After the patients were allowed to rest for ten minutes, they were switched to a suitable position to relax. Measurement was performed on left brachial artery in all patients. The transducer (7 MHz, Acuson 128 XP, Mountain view, CA) was placed 5 cm above the antecubital fossa, along the course where the artery could be best viewed longitudinally. In all patients, measurements were taken at rest and during reactive hyperemia (reactive hyperemia increases stress in the vascular wall, thereby leading to release of vasoactive mediators and flow-related vasodilation). During the measurement, simultaneous electrocardiography (ECG) recording was performed, and arterial lumen diameter was measured with ECG recording between *T* and *Q* waves. Lumen diameter was calculated as the distance between lumen and intima reflections of anterior and posterior walls. Measurement of lumen diameter at rest was repeated thrice for each patient, and the mean thereof was taken as basal value. Flow rate was measured and recorded via Doppler at the point of measurement of vein diameter during the peak of systole. After basal measurements were made, the ring-shaped of cuff the sphygmomanometer was placed on the brachial artery and inflated with a pressure of 250-300 mmHg so that the blood flow in the distal completely stopped and waited for five minutes. Then, the air inside the cuff was evacuated, and the diameter of the brachial artery was measured at the 1, 2, and 3 minutes. The highest value was taken as the reference value for the maximum dilation capacity and was used in calculation of FMD. FMD was defined as the ratio of the flow-mediated diameter increase observed in the vessel to the basal value and was calculated and recorded by the formula of “(maximum diameter basal diameter/basal diameter) × 100.” Flow rate was measured both in the basal state and at 15 seconds, where the flow rate was maximal, and the “hyperaemic flow ratio” was defined as the percentage of increase in the flow rate measured at 15 seconds compared to the basal value.

## 3. Statistical Evaluation

The data obtained from the study groups were compared statistically using SPSS 15.0 for Windows Evaluation Version package. In statistical analysis, distribution of measurable data was defined as arithmetic mean ± standard deviation. The difference between the two groups was evaluated using the Mann–Whitney *U* test. The relationship between the variables was calculated according to linear correlation coefficient. In all statistical comparisons, the significance level was accepted as *p* < 0.05.

## 4. Findings

**Figure 1:**
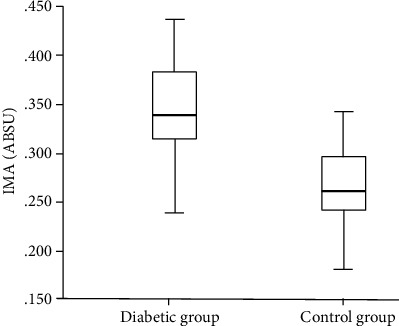
Ischemia-modified albumin (IMA) levels of diabetic patients as compared with the control group (*p* < 0.001).

**Figure 2:**
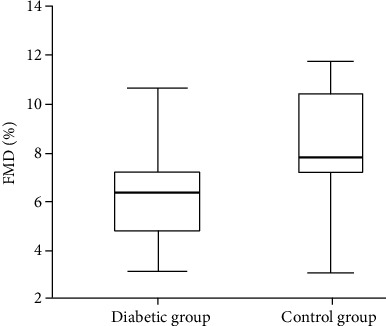
Distribution of FMD levels by groups.

**Figure 3:**
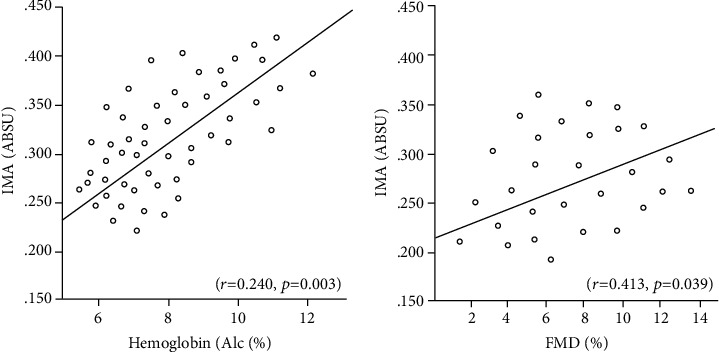
Correlation analyses of ischemia-modified albumin level with HbA1C and FMD in the whole diabetic patient group.

In the comparison between the diabetic group and the control group, no difference was observed in terms of age, BMI, presence of hypertension, smoking status, lipid parameters, and creatinine clearance (Table 1). SOD activity was significantly lower in the diabetic group than in the control group, and ET-1 was significantly higher (*p* < 0.001) (Table 1). IMA was significantly higher in the diabetic group than the control group (*p* < 0.001) (Table 1, Figure 1). FMD was significantly lower in diabetic groups compared to the control group (*p* < 0.002) (Table 1, Figure 2). Linear correlation test used for calculate correlation between IMA and various parameters in the diabetic ED group. IMA was positively associated with HbA1c (*r* = 0.240, *p* = 0.033) and FMD (*r* = 0.413, *p* = 0.039) (Table 1, Figure 3).

## 5. Discussion

In the early stages of diabetes, hyperglycemia leads to changes in blood flow and vascular permeability. It reduces nitric oxide (NO) release in the capillary region by increasing blood flow and intracapillary pressure. With the microvascular hypertension as well as the effect of endothelial and support cells, the gene expression occurs and thereby, irreversible microvascular occlusion process starts. This occlusion process in diabetic cells is also often accompanied by a loss of microvascular cells resulting from apoptosis [19]. In their comparative studies in diabetic and normal men, Ruzbarsky and Michal found intimal fibrous proliferation, medial fibrosis, and calcification as well as arterial lumen narrowed and/or occluded by thrombus in the cavernous artery [20]. Simopoulos et al. showed that there was a significant loss of total sinusoidal volume and vascular volume in patients with diabetes as compared to the control in a three-dimensional quantitative penile vascular analysis with X-ray microcomputer tomography [21].

ET-1, a vasoactive peptide produced in vascular endothelial and vascular smooth muscle cells, causes proliferation in smooth muscles in the vascular wall and thus triggering fibrosis and inflammation [22–26]. In the early stages of endothelial dysfunction, there exists an impaired balance between NO and ET-1, and vasorelaxation is disrupted [27, 28]. Among patients with diabetes, ET-1 was found to be higher in those with microalbuminuria, in those with high level of hemoglobin A1c, and in those with diabetic retinopathy [29, 30]. Studies have shown that ET-1 level is associated with glycemic control [31].

ET-1, released from endothelial cells, responsible for vascular tonus as a potent arterial and venous vasoconstrictor, also controls penile flaccidity during erection. In patients with diabetes, peripheral venous and cavernosal blood ET-1 levels were higher than patients with nondiabetes [32]. Sullivan et al. demonstrated that in patients with diabetes, the ET-1 receptor density is increased. On the other hand, it was reported that increased ET-1 release from cavernosal tissue endothelial cells might play a role in erectile functions with its direct contractile effect on smooth muscle [33].

In our study, ET-1, a biochemical marker for endothelial dysfunction, was determined to be higher in the diabetic group than the control group (*p* < 0.001).

Another method used in evaluation of endothelial functions is FMD. This noninvasive method is based on measuring the increased blood flow in the brachial artery due to the pressure created by the increased blood flow in the vascular wall [34–36].

There are publications in the literature reporting that endothelial dysfunction can be detected by FMD in patients without any microvascular complications. In a study by Sibal et al., 74 type 1 diabetes (T1 DM) patients without any microvascular complications were compared with 80 healthy controls [37]. FMD measurements were found to less in the T1DM group by 45% as compared to the control group, and the difference between the groups was reported to be significant. The authors suggested that abnormalities in endothelial functions as well as prothrombotic and proinflammatory condition existed far before the development of the clinically apparent arterial damage. In a study conducted by Vissoky et al. comprising 57 T1DM patients, it was reported that even in patients with newly diagnosed DM, endothelial dysfunction detectable by FMD existed regardless of age, smoking status, hyperlipidemia, and hypertension [38].

There are numerous studies in the literature that show endothelial dysfunction and reduced NO formation in ED [39]. Kaiser et al. compared 30 men with ED, but without a clear sign of vascular disease or risk factor with a similar control group without ED. There was no difference between the groups in terms of intima-medial thickness, coronary calcification, pulse wave velocity, or aortic distension. However, there was a significant reduction in FMD in men with ED [40]. This suggests that ED may be an early sign of vascular flow abnormalities and endothelial dysfunction.

In our study, we determined that FMD measurements, an indicator of endothelial dysfunction, were reduced in the diabetic group, which is in line with the literature (*p* < 0.002).

While it has been already known that endothelial dysfunction has an effect on the formation of erectile dysfunction (ED), with our study, it has been noted that the process takes a more serious dimension with the addition of diabetes to this association. It was found that the said effect incrementally increased in patients with diabetes and ED as compared to those with ED alone, and there was a significant difference in endothelial dysfunction between the two groups. The fact that the etiology of ED in patients with nondiabetes has not been examined in detail and therefore, we could not make an etiological evaluation with diabetic patients that can be seen as a limitation of our article.

Tissues sustain oxidative damage under the harmful effect of ROS, and this is more pronounced and intense in diabetes. Jones et al. found that basal superoxide (0_2_^−^) levels in diabetic rabbit model cavernosal tissue segments increased markedly and significantly compared to the control [41]. Owing to its increased concentration and activity of 0_2_^−^, NO undergoes a rapid breakdown, and NO-dependent vasodilation is disrupted. Superoxide dismutase (SOD) provides natural protection against NO breakdown. SOD protects NO activity by preventing ROS from forming peroxynitrite. With addition of SOD, a significant increase has been shown in significantly impaired NO as well as the nerve-dependent relaxation of smooth muscles in diabetic rabbit cavernosal muscle fibers compared to the control. In the diabetic rabbit model, it was thought that the significant increase in NOS binding sites was ascribable to substrate deficiency, impairment of NO bioavailability due to the advanced glycosylation end products (glycation end products) (AGEs), or NO inactivation by 0_2_^−^, and this event played a role in the pathogenesis of diabetic erectile dysfunction [41].

In our study, the activity of superoxide dismutase (SOD) which is an antioxidant enzyme was evaluated as well. While the SOD activity of the control group was 0.061 ± 0.014 U/ml, the SOD activity of the diabetic group was 0.031 ± 0.01 U/ml. This difference between the groups was thought to be statistically significant (*p* < 0.001). This result is similar to those obtained by many studies in the literature that were conducted by including both diabetes and SOD [42–45].

In patients with diabetes, albumin is modified in presence of chronic hypoxia formed due to oxidative stress and hyperglycemia, and serum IMA level increases [13, 46, 47]. Dayanand et al. studied the use of IMA, which is a new marker of tissue ischemia and oxidative stress, as a sign of ischemic changes in type 2 DM patients in a case-control study and concluded that high IMA levels in these patients might indicate an underlying subclinical vascular disease [48]. Furthermore, Kaefer et al. showed that there was a significant correlation between high IMA levels and fasting blood glucose, glycosylated albumin, and C-reactive protein in patients with type 2 diabetes [49].

In our study, IMA was found to be significantly higher in the diabetic group than the control group (*p* < 0.001). Our result is compatible with the literature, and higher levels of IMA in the diabetic group suggest that patients with diabetic erectile dysfunction are at higher risk for both atherosclerotic risk factors and oxidative stress than the nondiabetic group.

According to our findings, coexistence of erectile dysfunction and diabetes is a complex condition that includes endothelial dysfunction, oxidative stress, and tissue ischemia. With the correlation between the markers for each, we have once again demonstrated the seriousness of coexistence of erectile dysfunction and diabetes.

Method of reducing such ischemia and oxidative stress markers as IMA levels as well as other factors affecting endothelial dysfunction can be used in elaboration of new strategies to prevent diabetes and erectile dysfunction.

Our article was presented as an oral presentation at the 29th National Urology Congress on 17-21 November 2020 (https://www.uroturk.org.tr/urolojiData/uploads/files/29-uroloji-bildiri-kitabi.pdf).

## Figures and Tables

**Table 1:** General characteristics and biochemical parameters of diabetic patients in comparison with the control group.

	Diabetic group (*n* = 46)	Control group (*n* = 40)	*p*
Age (years)	51.5 ± 9.2	54.78 ± 12.2	NS
BMI (kg/m^2)^	31.6 ± 6.3	32.4 ± 8.4	NS
Smoking (*n*) (%)	12 (26%)	10 (25%)	NS
Hypertension treatment (*n*) (%)	26 (56%)	21 (52%)	NS
HDL (mg/dl)	47.88 ± 10.4	48.32 ± 11.2	NS
LDL (mg/dl)	124.2 ± 35.2	108 ± 34.3	NS
Triglyceride (mg/dl)	119.2 ± 52.4	107.3 ± 35.7	NS
Creatinine clearance (mg/min.)	117.3 ± 49.8	120.92 ± 30.4	NS
Fasting glucose level (mg/dl)	148.5 ± 50.2	86.9 ± 11.3	*p* < 0.001
Diabetes duration (years)	8.14 ± 4.8		
Hemoglobin A1c (%)	7.3 ± 2.5		
IMA (ABSU)	0.348 ± 0.042	0.263 ± 0.049	*p* < 0.001
SOD(U/ml)	0.031 ± 0.01	0.061 ± 0.014	*p* < 0.001
ET-1 (ng/ml)	0.28 ± 0.05	0.22 ± 0.04	*p* < 0.001
FMD (%)	6.4 (3.2-10.6)	7.9 (3.2-11.9)	*p* < 0.002

## Data Availability

The datasets used and/or analyzed during the current study are available from the corresponding author on reasonable request.
